# COVID-19 and Pregnancy: An Updated Review about Evidence-Based Therapeutic Strategies

**DOI:** 10.3390/jpm13071035

**Published:** 2023-06-23

**Authors:** Alessandro Favilli, Marta Mattei Gentili, Francesca De Paola, Antonio Simone Laganà, Amerigo Vitagliano, Mariachiara Bosco, Ettore Cicinelli, Vito Chiantera, Stefano Uccella, Fabio Parazzini, Sandro Gerli, Simone Garzon

**Affiliations:** 1 Section of Obstetrics and Gynecology, Department of Medicine and Surgery, University of Perugia, 06123 Perugia, Italy; martolamg90@gmail.com (M.M.G.); francescadepaola95@libero.it (F.D.P.); sandro.gerli@unipg.it (S.G.); 2 Unit of Gynecologic Oncology, ARNAS “Civico–Di Cristina–Benfratelli”, Department of Health Promotion, Mother and Child Care, Internal Medicine and Medical Specialties (PROMISE), University of Palermo, 90127 Palermo, Italy; antoniosimone.lagana@unipa.it (A.S.L.); vito.chiantera@unipa.it (V.C.); 3 Department of Biomedical and Human Oncological Science (DIMO), 1st Unit of Obstetrics and Gynecology, University of Bari, 70121 Bari, Italy; amerigo.vitagliano@gmail.com (A.V.); ettore.cicinelli@uniba.it (E.C.); 4 Unit of Obstetrics and Gynecology-Department of Surgery, Dentistry, Pediatrics, and Gynecology, AOUI Verona-University of Verona Piazzale A. Stefani 1, 37126 Verona, Italy; mariachiara.bosco@univr.it (M.B.); stefano.uccella@univr.it (S.U.); simone.garzon@univr.it (S.G.); 5 Department of Clinic and Community Science, Mangiagalli Hospital, University of Milan, 20122 Milan, Italy; fabio.parazzini@unimi.it

**Keywords:** COVID-19, therapy, pregnancy, pandemic, SARS-CoV-2

## Abstract

The COVID-19 pandemic posed a significant challenge for clinicians in managing pregnant women, who were at high risk of virus transmission and severe illness. While the WHO declared in May 2023 that COVID-19 is no longer a public health emergency, it emphasized that it remains a global health threat. Despite the success of vaccines, the possibility of new pandemic waves due to viral mutations should be considered. Ongoing assessment of the safety and effectiveness of pharmacological therapies is crucial in clinical practice. This narrative review summarizes the evidence-based therapeutic strategies for pregnant women with COVID-19, considering over three years of pandemic experience. The review discusses the safety and effectiveness of various drug regimens (antivirals, anticoagulants, corticosteroids, immunoglobulins, monoclonal antibodies, and therapeutic gases) and procedures (prone positioning and extracorporeal membrane oxygenation). Drugs with contraindications, inefficacy during pregnancy, or unknown adverse effects were excluded from our evaluation. The aim is to provide healthcare professionals with a comprehensive guide for managing pregnant women with COVID-19 based on lessons learned from the pandemic outbreak.

## 1. Introduction

In December 2019, a new severe acute respiratory syndrome coronavirus 2 (SARS-CoV-2) caused a serious public health threat worldwide. This highly contagious virus rapidly spread worldwide, starting from the Asiatic continent and specifically from Wuhan, China [1]. The World Health Organization (WHO) declared the Coronavirus disease 2019 (COVID-19) as a pandemic outbreak within a few months. It has been estimated that 5.9 million lives have been lost worldwide. However, recent analyses have speculated a number three times greater [2].

SARS-CoV-2 is responsible for a broad clinical spectrum of symptoms, ranging from asymptomatic infection or mild upper respiratory tract illness to severe viral pneumonia with respiratory failure [3,4,5]. Particularly at the beginning of the pandemic, when pathogenesis and therapy were unknown, and a specific vaccine was not yet available, thousands of people worldwide died due to COVID-19 as a direct consequence of interstitial lung disease [6]. In this scenario, managing pregnant women affected by COVID-19 represented a new challenge for midwives and obstetricians, requiring major efforts by healthcare professionals due to the lack of scientific evidence and the impossibility of rescheduling obstetrics care [7,8,9,10].

Pregnant women are a particular population at a higher risk of virus transmission and severe COVID-19, especially during the third trimester [1,11,12]. This susceptibility is related to the physiological suppression of the immune system that characterizes pregnancy and the changes in the cardio-pulmonary function that occur as a physiologic adaptation mechanism to the enlargement of the uterus and fetal development [13,14]. Moreover, assessing how managing pregnant women affected by COVID-19 was a long journey with several obstacles, such as the “protection by exclusion” issue: the ban of pregnant women, at least at the beginning, from potentially beneficial treatments. These limitations made it extremely difficult to test the safety and efficacy of drugs for COVID-19 in pregnancy [15,16,17,18]. Nevertheless, since the first months of the pandemic, therapeutic proposals have been developed, starting from pharmacological protocols already known to be safe in pregnancy and based on what was already known about previous coronavirus diseases (SARS-CoV-1 and MERS-CoV) [19,20].

Recently, after more than three years of pandemic outbreak, the WHO has declared that COVID-19 is no longer a “global health emergency” while stressing that it remains a global health threat [21,22]. Even though the “darkest hour” seems to have passed and the advent of vaccines has represented a breakthrough in the battle against SARS-CoV-2, physicians should be aware of the possibility of a new pandemic wave due to virus mutations [23]. In this regard, assessing the safety and effectiveness of pharmacological therapy remains a priority in clinical practice [24]. In fact, over the past two years, we have witnessed an extreme ability of the virus to mutate, resulting in the emergence of new variants [25,26]. On the other hand, the safety and effectiveness of both old and new therapeutic strategies have been validated relatively quickly and utilized for pregnant patients in clinical practice. [15,27,28]. Additionally, some drugs that were considered promising or beneficial are now clearly declared unhelpful in the treatment of COVID-19 disease in pregnancy.

This narrative review aims to provide an updated, evidence-based document outlining effective, safe, and tested therapeutic strategies for pregnant women affected by COVID-19.

## 2. Materials and Methods

This is a narrative review of published data on the effectiveness and safety of available treatments for COVID-19 in pregnant women. The review was reported and qualitatively assessed following the SANRA, the Scale for the Assessment of Narrative Review Articles [29].

We performed the research using a narrative review method [30]. Electronic databases (PubMed, Scopus, and Google Scholar) were searched until 17 February 2023 (without date restriction) for relevant publications in the English language focusing on, but not limited to, the use of the following key search terms: SARS-CoV-2, COVID-19, pregnancy, therapy. The electronic search and the eligibility of studies were independently assessed by two authors (MMG and FDP).

No restrictions on the study design were applied. Studies were selected if they provided helpful information on tested therapy and management of COVID-19 in pregnancy. Treatments deemed unsafe, useless, or unclear after two years of the COVID-19 pandemic were excluded. The first selection was based on the title, the second on the abstract, and the third on the full-text article. The bibliography was also analyzed to avoid missing potentially relevant publications. The most relevant articles for the purposes of this narrative review were included.

## 3. Results

Due to the nature of the findings, we opted for a narrative synthesis of the results from selected articles. Table 1 provides a summary of the drugs and procedures, with associated dosage and application, that have been reported to have valuable evidence of efficacy in the treatment of COVID-19 during pregnancy.

### 3.1. Drugs

#### 3.1.1. Antivirals

##### Nirmatrelvir/Ritonavir

Nirmatrelvir/Ritonavir is one of the first oral antiviral drugs used in the treatment of mild-to-moderate COVID-19 [31]; clinical studies suggest that it can lower the risk of severe COVID-19 by almost 90% [32]. A clinical trial by Hammond et al. [33] showed a reduction of hospital admission or death of 97% in adult patients affected by the Delta variant who underwent treatment with Nirmatrelvir/Ritonavir compared to placebo; laboratory studies and a large real-world retrospective study have also demonstrated the efficacy of this treatment against the Omicron variant and other SARS-CoV-2 variants [34,35].

The 13th updated recommendation of the World Health Organization (WHO) regarding the use of Nirmatrelvir/Ritonavir in pregnant and breastfeeding women with non-severe COVID-19 illness stated that there were no reported severe adverse events linked to the administration of this drug during pregnancy or breastfeeding neither for the mother nor children; however, there was residual uncertainty about the presence of undesirable effects. Therefore, the recommendation was that the use or non-use of Nirmatrelvir/Ritonavir in pregnant or breastfeeding women with non-severe COVID-19 should be determined through shared, fully informed decision-making between the mother and healthcare provider [36,37,38].

Both Nirmatrelvir and Ritonavir are protease inhibitors. Nirmatrelvir was developed by modifying an earlier drug, originally developed during the SARS-CoV-1 pandemic in 2002/03 [39]. Nirmatrelvir can be administered orally, and the recommended dose is two tablets, each containing 150 mg of Nirmatrelvir, plus one tablet containing 100 mg of Ritonavir twice a day for five days in non-hospitalized patients [40]. The administration should start as early as possible during the disease, ideally within five days of disease onset [41]. Side effects are uncommon (they may occur in less than one in ten individuals), and the most common ones include headache, diarrhea, taste disturbance (dysgeusia), and vomiting [33,40]. Nirmatrelvir/ritonavir is considered safe in pregnancy and breastfeeding, but it is not FDA-approved for any particular category of risk [42,43,44].

##### Remdesivir

Remdesivir was the first antiviral approved by FDA for use in adults and children affected by COVID-19. It works by inhibiting the RNA-dependent RNA polymerase, which is essential for viral replication [45]. Remdesivir was studied during the Ebola epidemic in central Africa, including trials involving pregnant women, with excellent results and no side effects on maternal and fetal health [46]. Regarding its efficacy and safety in pregnant patients with COVID-19 infection, most data come from case reports, case series, or observational studies with the administration of Remdesivir after the first trimester; these studies provided encouraging results of recovery rate and absence of adverse pregnancy outcomes [47,48]. Burwick et al. reported no side effects among the women treated in this study, and there were no fetal or maternal deaths related to the drug during the study [47].

The limited data suggest that Remdesivir is well tolerated in the last two trimesters of pregnancy, while evidence is less clear concerning the first trimester [45]. Remdesivir does not belong to any FDA category, as there are insufficient data on the use of this drug in pregnant women to inform a drug-related risk [49]. The recommended dosage for patients over 12 years and over 40 kg is a single dose of 200 mg IV on the first day, followed by 100 mg/daily for 5 to 10 days [50].

#### 3.1.2. Anticoagulant

##### Low Molecular Weight Heparin

Heparin is a glycosaminoglycan that works by preventing the formation of clots and the extension of existing clots within the blood. It binds and activates the enzyme inhibitor antithrombin III (AT), which then inactivates thrombin, factor Xa, and other proteases, thus inhibiting coagulation. Due to its heavy molecular weight, heparin does not cross the placental barrier, and its use is considered safe during pregnancy and breastfeeding [18].

Since the beginning of the COVID-19 pandemic, it has been well-known that affected patients are at high risk of developing thromboembolic complications [51]. Considering that pregnant women are already in a prothrombotic condition, low molecular weight heparin (LMWH) prophylaxis with 4000 UI per day is recommended for all affected women admitted with confirmed or suspected COVID-19 infection. The exception is women expected to give birth within 12 h. After delivery, LMWH should be administered for at least 10 days, regardless of the modality of delivery [52].

There are considerable differences in clinical practice around the world: some authors suggested doubling the typical dose of prophylactic heparin (e.g., Enoxaparin 4000 UI twice daily, rather than once daily) and the duration varies between 7–14 days up to 6 weeks postpartum depending of the presence of risk factors for venous thromboembolism (VTE) [53,54].

Therefore, administering heparin to a pregnant and postpartum woman with COVID-19 should be determined by considering the severity of the infection, comorbidities, proximity to delivery, and whether the woman is in hospital care or at home [55]. Heparin is not assigned to any FDA category of risk [56].

#### 3.1.3. Corticosteroids

There is considerable disagreement among guidelines about whether corticosteroids should be given and when they should be recommended [57]. The appropriate corticosteroid for a pregnant patient depends primarily on whether the treatment is intended for the mother or the fetus.

Since the beginning of the pandemic, there has not been a significant change in the use of corticosteroids for fetal pulmonary maturity in pregnant patients who test positive for COVID-19. Short courses of Betamethasone (2 doses of 12 mg i.m. 24 h apart) and Dexamethasone (4 doses of 6 mg i.m. 12 h apart) are commonly used in pregnancy from 24 + 0 to 33 + 6 weeks of gestation, taking into account the higher risk of preterm prelabour rupture of membranes (pPROM) and subsequent preterm delivery in women with COVID-19, to insure pulmonary maturity of the fetus [27,58].

For pregnant patients with suspected or confirmed COVID-19 at 34 + 0 to 36 + 6 weeks of gestation and at risk of preterm birth within 7 days, ACOG has advised against administering a course of Betamethasone due to unclear benefits to the neonate. However, this decision may need to be individualized, weighing the neonatal benefits with the risks of potential harm to the pregnant patient [59,60,61].

Pregnant patients affected by severe forms of COVID-19 infection who require oxygen supplementation or ventilatory support should receive systemic corticosteroids; according to RCOG guidelines, oral Prednisolone or intravenous Hydrocortisone for 10 days or until hospital discharge, whichever is sooner, can be used [62]. If steroids are not indicated for fetal lung maturity, oral Prednisolone 40 mg daily (or oral Methylprednisolone 32 mg daily) or IV Hydrocortisone 80 mg twice daily for 10 days or until discharge may be used. Conversely, if steroids are indicated for fetal lung maturity, intramuscular Dexamethasone 12 mg twice (24 h apart), immediately followed by oral Prednisolone 40 mg once a day or IV Hydrocortisone 80 mg twice daily, to complete a total of 10 days or until discharge, whichever is sooner [63].

Dexamethasone is registered in FDA category C. Bethametasone is not registered in any US FDA category. Prednisolone is registered in FDA category C/D; Hydrocortisone and Methylprednisolone are in FDA category C [64,65,66].

#### 3.1.4. Convalescent Plasma

The use of convalescent plasma (CP), also known as hyperimmune plasma, for treating COVID-19 has been widely studied. The mechanism of action of hyperimmune plasma is based on its ability to remove high-weight molecules from the blood, including antibodies, cytokines, and fibrin degradation products [67]. Convalescent plasma was first used in the late 19th century, even before the discovery of antibiotics, to treat several infectious diseases [68].

In the early stages of the COVID-19 pandemic, hyperimmune plasma was proposed as a treatment due to its benefits during SARS, MERS, and Ebola epidemics [69,70]. There are several case reports of pregnant women with COVID-19 being treated with hyperimmune plasma. Specifically, in these case reports, the patients were affected by a severe infection and required oxygen to maintain acceptable saturation [67,68]. As reported by the first systematic review of the literature regarding the treatment of COVID-19 with CP in pregnancy, the treatment was administered to twelve pregnant women affected by severe and mild Acute Respiratory Distress Syndrome (ARDS); in this group, two women had one comorbid condition (obesity), and five had multiple comorbid conditions. The result was that in about half of the cases, two CP units were required to obtain a clinical improvement, and the transfusion of the first CP unit was performed at a median of 2 days after admission to the hospital. Early infusion is essential to obtain the best antiviral effect of hyperimmune plasma, as documented by a recent randomized controlled trial [69]. Furthermore, a low rate of adverse effects of CP transfusion was recorded [71,72]. Additionally, an exploratory analysis of 4330 patients showed no significant difference in 7-day mortality between patients who received high-titer plasma and those who received low-titer plasma in the overall population [70].

The administration of hyperimmune plasma in patients affected by SARS and MERS resulted in some side effects, particularly cases of ARDS Transfusion Related Lung Injury (TRALI) [73,74]. In contrast, no side effects were observed in COVID-19 patients who received hyperimmune plasma [69].

Despite these encouraging findings, more evidence is required, ideally through randomized trials, to establish the administration of hyperimmune plasma as a safe and effective treatment for pregnant women with COVID-19. Indeed, according to Simonovich’s randomized study, the use of convalescent plasma did not yield significant clinical benefits compared with placebo in patients with severe COVID-19 pneumonia [70].

#### 3.1.5. Monoclonal Antibodies

##### Tocilizumab

It is a humanized monoclonal antibody that blocks interleukine-6 membrane receptors, whose levels are exceptionally high during COVID-19 infection [75]. Tocilizumab is licensed in over 75 countries for rheumatoid arthritis and related rheumatological diseases [76].

Tocilizumab was the first antibody proposed as a therapy for patients with severe respiratory failure due to COVID-19 [77]. The recommended adult dose is 8 mg/kg administered by IV infusions, to a maximum of 800 mg. If there is no improvement after the first dose, it is possible to administer a second dose after at least 8 h [78,79].

Due to the exclusion of pregnant women from clinical trials, little evidence is available about the administration of this drug during pregnancy. Abdullah et al. cited the case of two pregnant women in the last trimester of pregnancy who received Tocilizumab for a severe form of respiratory failure related to COVID-19. Both women experienced an immediate improvement in respiratory conditions without any fetal consequences, as reported during follow-up [75]. Moreover, in Jorgensen’s review, Tocilizumab administration was reported in several cases during the third trimester and in critically ill pregnant patients, and all pregnancies resulted in live births, but most had limited neonatal follow-up [76]. There was one case of maternal cytomegalovirus reactivation and congenital infection [80]. A dose-related increase in the incidence of secondary opportunistic infections was identified among patients receiving long-term Tocilizumab in rheumatoid arthritis trials [81]. Luckily, this has not been observed in clinical trials of Tocilizumab for COVID-19 [82].

It is mandatory to carefully evaluate each case in a multidisciplinary manner to avoid excluding pregnant women with severe forms of COVID-19 from the benefits of this treatment. However, it should be considered that Tocilizumab can cross the placenta, especially when administered during the third trimester, and may slightly increase the risk of preterm delivery. Nevertheless, no fetal effects have been observed [78,79,83,84].

The FDA has approved the use of intravenous Tocilizumab to treat COVID-19 in hospitalized adult patients receiving systemic corticosteroid treatment and requiring supplemental oxygen or mechanical ventilation [85].

#### 3.1.6. Nitric Oxide

It was recently proposed as adjunctive therapy for patients with severe COVID-19 infection who need oxygen therapy and mechanical ventilation [86]. Nitric oxide (NO) was first approved by the FDA in 1999 for use in neonates with severe respiratory failure associated with pulmonary hypertension requiring mechanical ventilation [87]. Recent evidence shows that NO can be used in pregnant women without any fetal or maternal consequences. In particular, due to its specific ability to selectively cause pulmonary vasodilatation, the administration of this gas seems to reduce the need for oxygen and hospitalization duration [86,88]. According to a 2022 study by Valsecchi et al., reducing the need for oxygen is crucial for pregnant patients with COVID-19 because pregnancy itself increases oxygen demand due to the low placental oxygen tension [86]. Akaberi et al. demonstrated that nitric oxide also has an antiviral effect by inhibiting the SARS-CoV-2 spike protein [89]. However, due to a limited number of studies, which are mostly retrospective, and a limited number of patients enrolled, a clear therapeutic strategy for using NO in pregnancy has yet to be established. In the meantime, caution should be exercised in using NO in pregnant women with COVID-19.

### 3.2. Procedures

#### 3.2.1. Extracorporeal Membrane Oxygenation (ECMO)

Extracorporeal membrane oxygenation (ECMO) is a rescue therapy used in severe cases of COVID-19-related ARDS, with careful evaluation of the risk–benefit balance in a multidisciplinary team. Pregnancy per se is not a contraindication, but the enlargement of the uterus and the increased oxygen demand due to the higher cardiac output can make the cannulation site more challenging. Usually, the jugular vein is preferred even if it is smaller [90].

During the ECMO procedure, there is a higher risk of bleeding, so it is recommended to avoid anticoagulants in pregnant women who need to deliver during the procedure [91,92]. Barrantes et al. described a case series of pregnant and postpartum women with severe COVID-19 and ARDS treated by ECMO, reporting excellent maternal and neonatal survival rates. All patients were successfully decannulated from ECMO and discharged from the hospital, and none of the followed-up newborns had laboratory or clinical evidence of COVID-19 [93]. Nevertheless, a case report published in 2022 reported some adverse effects of the ECMO procedure, such as a higher risk of thromboembolic events, infectious disease, and neonatal mortality due to respiratory causes [94]. Another case series of five patients who underwent ECMO because of respiratory failure reported a lower survival rate, underlining the fact that ECMO in the third trimester is feasible but not free of complications [95]. For these reasons, even though the procedure has good safety and efficacy in prolonging pregnancy in patients with COVID-19, we must keep in mind that patients who undergo this treatment are in severe clinical conditions. Therefore, it is mandatory to carefully balance risks and benefits in each case to try to avoid or minimize serious adverse effects [94,96,97].

#### 3.2.2. Prone Positioning

In the presence of ARDS of any etiology, prone positioning has been proven to benefit oxygenation and mortality; recent global anecdotal reports have found prone positioning particularly helpful for patients with COVID-19 and moderate or severe respiratory disease [98]. Although data are limited, case reports and expert experience suggest that prone positioning may be particularly useful in pregnant patients owing to its ability to relieve both diaphragmatic compression from abdominal contents and aortocaval compression from the gravid uterus if performed correctly [99]. Prone positioning is an opportunity to improve oxygenation in patients with severe hypoxemia when the next consideration is the delivery of a premature infant or maternal cannulation for extracorporeal membrane oxygenation [100]. Obviously, prone positioning for patients at 34 weeks of gestation or more may be more technically challenging owing to the gravid uterus at advanced gestational ages, and the risks and benefits of delivery before prone positioning should be strongly considered. Furthermore, special attention must be paid to avoid aortocaval compression [98]. Wong et al. reported thirteen pregnant patients affected by mild and severe forms of COVID-19 who had prone positioning during their hospitalization. No prone positioning sessions were terminated urgently due to maternal hemodynamic instability, worsening oxygenation or ventilation, or fetal intolerance. In only one patient, uterine contractions while prone led to repositioning supine, after which the contractions resolved; the patient was maintained prone subsequently without issue. On some occasions, difficulty maintaining continuous fetal monitoring while in the prone position required an earlier return to the supine position [101].

Overall, favorable maternal and fetal survival were observed in obstetric patients with severe COVID-19 requiring mechanical ventilation.

**Table 1:** Relevant data of drugs and procedures analyzed.

Drug Category	Active Principle	Mechanisms of Action Against SARS-CoV-2	Dosage	FDA Category	Efficacy	References
Antivirals	Nilmaterlvir/Ritonavir	Protease Inhibitor/Protease Inhibitor	300 mg/100 mg every 12 h for 5 days	Not assigned	++	[31,32,33,34,35,36,37,38,39,40,41,42,43,44]
	Remdesivir	RNA polymerase inhibitor	200 mg IV the 1st day followed by 100mg daily for 5–10 days	Not assigned	+	[45,46,47,48,49,50]
Anticoagulants	Low Molecular Weight Heparin	Binds and activates Antithrombine III	4000UI daily, duration varies depending on risk factors	Not assigned	++	[18,51,52,53,54,55,56]
Corticosteroids	Bethametasone	Mitigation of host inflammatory response; prevention of neonatal respiratory distress syndrome	12 mg IM 24 h apart	Not assigned	++	[57,58,59,60,61,62,63,64,65,66]
	Dexamethasone	Mitigation of host inflammatory response; prevention of neonatal respiratory distress syndrome	6 mg IM 12 h apart for 4 times	C	++	
	Methylprednisolone	Mitigation of host inflammatory response	32 mg daily orally for 10 days or until discharge	C	++	
	Prednisolone	Mitigation of host inflammatory response	40 mg daily orally for 10 days or until discharge	C/D	++	
	Hidrocortisone	Mitigation of host inflammatory response	80 mg IV twice a day for 10 days or until discharge	C	++	
Immunoglobulins	Convalescent Plasma	Removing high-weight molecules from blood, including antibodies, cytokines, fibrin degradation products	200–500 mL I.V. once; varies among studies	/	+	[67,68,69,70,71,72,73,74]
Monoclonal Antibodies	Tocilizumab	Blocking interleukine-6 membrane receptors	8 mg/kg IV infusion (max 800 mg); second dose possible after 8 h.	Not assigned	++	[75,76,77,78,79,80,81,82,83,84,85]
Therapeutic gas	Nitric Oxide	Inducing selective pulmonary vasodilatation; blocking SARS-CoV-2 spike protein.	10–80 ppm for 30 min twice daily by snug-fitting mask	C	+	[86,87,88,89]
**Procedures**		**Mechanisms of Action Against SARS-CoV-2**	**Application**	**FDA category**	**Efficacy**	**References**
ECMO		Drainage of blood, diversion to membrane system and returning to patient, providing respiratory/hemodynamic support	Duration depends on patients’ response	/	+	[90,91,92,93,94,95,96,97]
Prone Positioning		Relieving diaphragmatic compression from abdominal contents and aortocaval compression from gravid uterus	At least 16 h per day in severe ARDS; 2 h in awake pregnant women with mild ARDS	/	+	[98,99,100,101]

Legend: ++: Drugs/procedures that have been shown to be safe and effective in pregnant women affected by COVID-19. +: Drugs/procedures that have been shown to be reasonably safe and effective in pregnant women affected by COVID-19, but more clinical evidence is needed.

## 4. Discussion

Our aim was to provide an overview of the standardized therapies currently used worldwide for the management of COVID-19 in pregnant patients. Treatments that have been deemed unsafe, useless, or unclear after 2 years of the COVID-19 pandemic are excluded from this review.

During the pandemic, a growing body of evidence, mostly from retrospective studies, case reports, and case series, has helped clinicians around the world care for this special group of patients. However, pregnant women’s exclusion from clinical trials has resulted in a lack of robust evidence to establish safe and valid guidelines specifically for pregnant women affected by COVID-19 disease [17,102]. Additionally, the advent of vaccines has reduced the urgency of finding new safe and effective treatments for COVID-19 in pregnant and breastfeeding women. Nevertheless, we now have enough knowledge to confirm that certain drugs can be reasonably excluded from treatment during pregnancy, while others have been shown to be effective and safe.

Since the beginning of the COVID-19 pandemic, several different molecules have been used based on their in vivo or in vitro activity against other viruses. For example, anti-malaria drugs such as chloroquine/hydroxychloroquine are considered safe during pregnancy and are used in areas where malaria is endemic. Initially, these molecules were used for their known antiviral and immunomodulatory activities [18], but due to a lack of clear benefits in patients affected by COVID-19, these drugs are no longer recommended for COVID-19 treatment. The US FDA revoked authorization to use these agents in patients with severe COVID-19 because the benefits do not outweigh the risks [103].

Antibiotics require a special mention, considering their widespread use in the early stages of the pandemic. A study conducted in Wuhan reported that 71% of patients were treated with antibiotics, but only 1% experienced coinfection with bacteria [5,104]. Several trials have been performed to address their effectiveness, most of which have evaluated the effects of Azithromycin. A meta-analysis by Popp et al. examined the efficacy and safety in out- and in-patient randomized controlled trials (RCTs) in which antibiotics were compared to non-treatment, placebo, standard care, or other treatment intervention with proven efficacy for COVID-19. They reported no reduction in the risk of death in hospitalized patients after 28 days of Azithromycin, and no benefits were observed in moderate/severe disease among in-patients [105]. Reports indicate that 3.5% of pregnant patients who presented COVID-19 had bacterial coinfection at hospital admission. Even though current evidence on bacterial infections in COVID-19 patients is limited, it supports the restrictive use of antibiotics, especially at admission [106]. Performing samples and cultures with antibiograms before giving any antibiotics is recommended to administer targeted therapy, thus improving the chances of healing and reducing the risks of resistance [107,108]. In addition to the global threat of antimicrobial resistance [109], there are other factors that we should consider before giving antibiotics to pregnant patients. Some antibiotics, like most drugs, enter the fetal circulation across the placenta and could cause fetal malformations or other alterations, depending on the time of exposure to the drug [110]. These alterations can affect maternal and fetal microbiota, leading to several diseases, including neurodevelopmental impairment, immunological, allergic and metabolic diseases, inflammatory bowel disease, and other infections in the offspring [111,112]. Azithromycin and Doxycycline were initially recommended due to their in vitro activity against viruses like Influenza and Dengue [113]. Azithromycin and Ceftriaxone are the most used antibiotics in pregnant patients due to their accessibility, low cost, and safety [107].

During the COVID-19 pandemic, new insights and knowledge were gained about monoclonal antibodies. Eculizumab, a monoclonal antibody used mainly for the treatment of atypical hemolytic uremic syndrome (SEUa), myasthenia gravis, and optic neuromyelitis, has been tested for severe forms of COVID-19 in adults [114,115,116,117]. In pregnant patients, a considerable improvement in clinical conditions with no need for mechanical ventilation and no adverse effects on the mothers or fetuses were observed [114]. However, the lack of data about its use in pregnant patients with COVID-19 represents a limitation, and more studies are needed to test the safety and effectiveness of this drug.

Casivirimab/Imdevimab is another monoclonal antibody used to treat severe forms of COVID-19 disease, especially during the Delta variant phase. In this case, no fetal adverse effects nor alterations in a fetal heartbeat were reported, and fever and cutaneous rash were the only symptoms due to the administration of the drug, according to the National Institute of Health [118,119]. However, data about the administration of Casirivimab/Imdevimab are still insufficient to considerate it wholly safe and riskless in pregnant women.

The real breakthrough in the fight against COVID-19 came with the advent of vaccines. The first COVID-19 vaccine became available under emergency-use authorization in the United States on 11 December 2020 [120]. The lack of safety and efficacy data on COVID-19 vaccination in pregnant people initially caused global vaccine skepticism in this population, with top health organizations giving mixed messages and recommendations [121]. Only in February 2022, the WHO assessed that the benefits of vaccination in pregnant people outweigh the potential risks [122]. Numerous studies have shown that vaccinated pregnant people have lower rates of COVID-19 infections [120,123], a lower chance of developing severe symptoms with a lower need for invasive therapies in case of infections, and lower mortality rates [100,120,123,124]. Regarding pregnancy outcomes, several studies reported no association of vaccination status with preterm birth, stillbirth, small for gestational age, very low birth weight, infant mortality, or neonatal hospitalizations [125,126,127,128]. In particular, data following vaccination during the first trimester of pregnancy showed no difference in the prevalence of miscarriage, fetal malformation, and pregnancy outcome compared with the general population [129,130]. Moreover, COVID-19 vaccination during pregnancy can protect the baby after birth through IgG antibodies that cross the placenta, especially if the vaccine is given during the third trimester [131,132].

## 5. Conclusions

The management of COVID-19 in pregnancy requires a personalized approach, taking into account the scientific evidence as well as the specific characteristics of each individual case. In addition, it is now widely recognized that the management of COVID-19 in pregnancy should involve a multidisciplinary team, including obstetricians, physicians, anesthetists, hematologists, and intensivists. This holistic approach is essential to make informed decisions based on the available evidence and to determine the best course of action for each pregnant woman affected by COVID-19, allowing a thorough assessment of the patient’s condition, consideration of the potential risks and benefits associated with therapy, and the development of a tailored management plan.

Many drugs have been tested, and significant steps have been made in the fight against the virus worldwide. Antivirals, anticoagulants, and corticosteroids remain essential in treating pregnant patients with COVID-19. Other therapies such as convalescent plasma, monoclonal antibodies, and ECMO have proved to be valuable and safe in severe cases of disease with promising results; thus, it is essential to collect more data, possibly coming from clinical trials, about this category of patients.

Vaccination remains the real first line of effective prevention that should be followed. Overall, many studies and articles on COVID-19 vaccination and pregnancy indicate that vaccines are safe and effective, so it is imperative to encourage pregnant women to get vaccinated.

## Data Availability

Not applicable.
